# Book Review: A Talent for Friendship: Rediscovery of a Remarkable Trait

**DOI:** 10.3389/fpsyg.2017.00641

**Published:** 2017-04-26

**Authors:** Gregory Bonn

**Affiliations:** General Studies, King Fahd University of Petroleum and MineralsDhahran, Saudi Arabia

**Keywords:** evolution, cooperation, friendship, human nature, native populations

In this wide-ranging work Professor John Terrell argues that friendship, or the cultivation of social ties outside the constraints of family and day-to-day necessity, is a defining characteristic of the human species. Terrell, an anthropologist who has done extensive fieldwork among native populations in New Guinea and surrounding regions, begins by describing his own explorations of the interpersonal networks that exist among the peoples indigenous to this region. He then gradually develops his “friendship hypothesis” through a review of findings ranging from psychology, cognitive neuroscience, evolutionary science, and anthropology, to the occasional forays into history and philosophy.

Terrell's primary argument critiques common, often implicit, assumptions underlying much evolutionary, psychological, and anthropological thought that portray human beings as fundamentally aggressive and self-centered. Mixing numerous anecdotes from his own field work, with a thorough review of findings from other fields, he builds his argument: those living in what most of us see as primitive conditions are far from the savages that they are assumed to be. He describes extensive networks of cooperation and sharing of resources among native peoples that have existed since pre-historic times, and have been passed along from generation to generation over the millennia. Such networks have survived despite intrusions by more “advanced” cultures as well as natural disasters that periodically led to the destruction and/or relocation of many of these settlements. In contrast to what would be predicted by darker visions of human nature, communities were not swallowed up or consumed by their neighbors when faced with challenges. These groups persisted and thrived, not despite of, but often assisted by other communities to which they had generations-old ties.

In support of his own anthropological observations, Terrell draws heavily upon work from other fields. He incorporates, for example, the work of mathematical biologist Martin Nowak (e.g., Nowak, 2006), as well as neurological findings related to Social Baseline Theory (e.g., Beckes and Coan, 2011). Which both suggest that human cooperative instincts have been central to our evolutionary success. They show how cooperation, throughout the history of our species has allowed us to minimize risk, and maximize the efficiency of our productive capabilities. Humans, the evidence shows, are by nature neurologically adapted for bonding with and receiving emotional comfort from others. Humans are affectively tuned, it is argued, so that a baseline level of emotional comfort depends upon being embedded within familiar surroundings. As Social Baseline Theory describes it, the ecological environment that humans are primarily adapted to is the company of other humans.

Terrell's other major theoretical point relates to the nature of human cognition, which he conceptually divides into three major divisions: The first two essentially correspond to system 1 and system 2 as described by Kahneman and others (i.e., fast and intuitive thinking vs. deliberative and analytical processing). The third cognitive system consists of the arguably unique human ability to think counterfactually, or to imagine situations that do not currently exist and have not been experienced. Terrell argues that this ability has provided humans with the ability to redesign our surroundings to a degree unknown elsewhere in the animal kingdom. However, this ability to think counterfactually, in Terrell's view, is also the source of the great conundrum of human existence: The fact that each of us is able to retreat into our own private worlds, and therein construct alternative realities and visions of what could or should be, means that we often find ourselves in the situation where our visions of how the world *ought* to be are in conflict with those of others. Terrell proposes then, that human conflict, rather than being rooted in a fundamentally violent and selfish nature, stems from differing internal visions of the ideal world and how best to manipulate reality.

It is on this rather simplistic notion that many, including myself to a point, will tend to disagree with Professor Terrell. Although, my own research's most consistent finding is that, across cultures, people's highest priorities relate to the formation and maintenance of enduring relationships (e.g., Bonn and Tafarodi, 2013; Bonn and Tam, 2016). I think that it is naïve to claim that humans, like all animals, are not hard-wired to act aggressively *in some situations*. Particularly when there is a perception that survival is threatened, humans can be quite willing and able to act in extremely brutal ways. Chalking up the many atrocities committed throughout human history to differences of opinion denies, I think, an important part of our emotional makeup. Affective neuroscience research indicates that humans, like all mammals, possess hard-wired rage responses, in addition to similarly hard-wired (and thankfully, for the most part much more active) drives toward nurturance and caring for others (see, for example Panksepp and Biven, 2012). In fairness, however, Terrell doesn't overstate his case too greatly by altogether dismissing human tendencies toward aggression and violence. His point is more that (despite the fact that mass slaughter is much more dramatic), as a general rule, humans historically have spent much, much more of their time and energy cooperating and helping each other succeed. If we hadn't, we most certainly wouldn't have come this far as a species.

Overall, I found this book to be both entertaining and thought provoking. For those of us who spend more than our share of time focusing on narrowly on specific topics, Professor Terrell provides uncommon breadth of perspective as well as fascinating real-life experiences: most readers will not be able to avoid broadening their horizons to some degree. His peripatetic style of writing is also, for the most part, quite entertaining. The numerous anecdotes and digressions, will, if you are a curious person, keep you easily engaged (although you may at times wonder where he is heading). I would recommend this book to anyone who has an interest in human nature, evolution, and the foundations of social life as well as to those who are looking for an alternative to the negativity and tribalism that permeates much public discourse recently.

## Author contributions

The author confirms being the sole contributor of this work and approved it for publication.

### Conflict of interest statement

The author declares that the research was conducted in the absence of any commercial or financial relationships that could be construed as a potential conflict of interest.
